# Aberrant expression of HIF3A in plasma of patients with non‐small cell lung cancer and its clinical significance

**DOI:** 10.1002/jcla.23889

**Published:** 2021-07-10

**Authors:** Liang Wei, Na Yuan, Yingying Chen, Pingping Gong

**Affiliations:** ^1^ Department of Thoracic Surgery The No.8 People’s Hospital of Qingdao Qingdao China

**Keywords:** HIF3A, hypoxia, mRNA gene, non‐small cell lung cancer, plasma

## Abstract

**Background:**

Hypoxia‐inducible factors (HIFs) have been evaluated in various cancers and diseases. However, the specific role of hypoxia‐inducible factor 3 alpha (HIF3A) in non‐small cell lung cancer (NSCLC) remains controversial.

**Materials and Methods:**

We investigated HIF3A mRNA expression in the plasma and tumor tissues of patients with NSCLC and explored its clinical significance. Plasma samples from 103 cases of lung adenocarcinoma (LUAD) and 96 cases of lung squamous cell carcinoma (LUSC), and tumor‐adjacent normal tissues from 58 LUAD and 62 LUSC cases were retrospectively evaluated at the No.8 People's Hospital of Qing Dao. HIF3A expression was explored using RT‐qPCR. The clinical significance of HIF3A was evaluated in the plasma and tumor tissues using the receiver operating curve (ROC) and the area under the curve (AUC).

**Results:**

Hypoxia‐inducible factor 3 alpha expression was notably downregulated in the plasma or tumor tissues of patients with LUAD and LUSC, compared with the healthy control group or adjacent normal tissues. Furthermore, HIF3A expression had a significant positive correlation in the plasma and tumor tissues of LUAD and LUSC patients. Meanwhile, the ROC‐AUCs achieved a significantly higher range, from 0.84 to 0.93, with the plasma or tumor tissues of NSCLC patients. Thus, HIF3A expression was not only correlated with plasma and tumor tissues, but also showed potential significance in NSCLC.

**Conclusion:**

Hypoxia‐inducible factor 3 alpha is aberrantly detectable in NSCLC patients in the plasma and tumor tissues. HIF3A may be involved in hypoxic responses during the development and occurrence of NSCLC.

## Graphical abstract

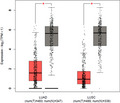

## 1 INTRODUCTION

Lung cancer is one of the most common malignant tumors and the primary cause of cancer‐related deaths worldwide.^1^, ^2^ There are two main types: small cell lung cancer (SCLC) and non‐small cell lung cancer (NSCLC). NSCLC is mainly characterized by lung adenocarcinoma (LUAD), lung squamous cell carcinoma (LUSC), and lung large cell carcinoma (LULC).^3^ Even though considerable progress has been observed in recent years in the development of diagnostic and prognostic biomarkers and anti‐cancer‐based therapeutics including chemotherapy, radiation therapy, biological targeted therapy, and immunotherapy, the mortality of patients with advanced NSCLC persists due to the inability to diagnose to condition at early stages or treat advanced conditions surgically.^2^, ^3^ Consequently, research into novel diagnostic biomarkers, target genes, and therapeutic target structures of NSCLC has attracted the attention of researchers.

Hypoxia‐inducible factors (HIFs) have been evaluated in various cancers and diseases.^4^ HIFs are heterodimers that include HIF α (oxygen‐labile) and HIF *β* subunits.^4^, ^5^ Three distinct HIF genes are found in humans and other vertebrates, but HIF signaling is acquired from variants of HIF‐1 α and HIF‐2 *β*.^6^, ^7^, ^8^, ^9^, ^10^ Meanwhile, the functions of the HIF‐3 complex are because of existing alternative spliced variants of HIF3A (hypoxia‐inducible factor 3 alpha, also known as HIF‐3 α), which result in four dissimilar variants of mRNA that code for additional isoforms by using different promoters.^11^ The effect of suppression on HIF‐1 and HIF‐2 activity is represented by the factors embracing the α‐3 subunit, which are anti‐regulatory for hypoxia‐induced expression of genes.^12^, ^13^

There is much to discover about HIF3A compared with HIF1A and HIF2A. Previous studies have discovered the tumor‐suppressive activities of HIF1A and HIF2A in renal cell carcinoma and lung cancer.^4^ However, the specific role of HIF3 in NSCLC remains controversial. The current study aimed to evaluate HIF3A mRNA expression in the plasma and tumor tissues of patients with LUAD and LUSC. Furthermore, this study aimed to evaluate the diagnostic value of HIF3A in NSCLC.

## 2 MATERIALS AND METHODS

### 2.1 Patients

Plasma samples from 103 cases of LUAD and 96 cases of LUSC, and tumor‐adjacent normal tissues from 58 cases of LUAD and 62 cases of LUSC were retrospectively collected from patients undergoing primary tumor resection surgery at the No.8 People's Hospital of Qing Dao. Paired plasma samples were also collected from the 58 LUAD and 62 LUSC patients from whom tissue samples were obtained. The patients were recruited from May 2019 to September 2020. All patients signed informed consent forms during their hospital stay. The study was approved by the ethics committee of the No.8 People's Hospital of Qing Dao and was conducted following the guidelines from the Declaration of Helsinki.

### 2.2 RNA isolation

Total RNA was extracted using phenol‐chloroform solutions and then homogenized using guanidine isothiocyanate (Trizol RNA Preparation kit; Invitrogen). RNA concentration was evaluated using a NanoDrop spectrophotometer ND1000 (NanoDrop Technologies Inc.).

### 2.3 RT‐qPCR

The total RNA in the plasma and tissue samples after transfection was extracted with TRIzol reagent, according to the manufacturer's instructions. Thereafter, the total RNA was reverse‐transcribed into cDNA using a reverse transcription kit (Shanghai Sangon Biological Engineering Co., Ltd.). The primers used were as follows: HIFA3,5′‐F: AGGCGCCAGAGGCACCATGGAC‐3′, R: 5′‐CATCCTGTGCGTTGGCTGCC‐3′,U6 F: 5′‐GAAGGTGAAGGTCGGAGTC‐3′, R: 5′‐GAAGATGGTGATGGGATTT‐3′. The PCR reaction conditions were as follows: A: pre‐denaturation at 95°C for 10 min, B: denaturation at 95°C for 15 s, annealing at 60°C for 15 s, and elongation at 72°C for 20 s, for a total of 40 cycles; and C: 72°C for 15 min. The reaction was terminated at 4°C. Three replicates were set for each sample, and 2^−△△Ct^ was used for the relative quantitative analysis of the data.

### 2.4 Bioinformatics

Bioinformatics were performed using GEPIA 2(http://gepia2.cancer‐pku.cn/). The data used were from TCGA normal and GTEx data.

### 2.5 Statistical analysis

SPSS 20.0 (SPSS Inc.) was used for all statistical analyses in the study, and data were expressed as mean ± SD. Clinical information of the patients in Tables 1 and 2 was analyzed by chi‐square test. A *t* test was performed to compare the two groups, and one‐way ANOVA was used to compare multiple groups. Pearson's correlation was used for the correlation analysis. The clinical significance of HIF3A was evaluated in plasma and tumor tissues using the receiver operating curve (ROC) and the area under the curve (AUC). Statistical significance was set at *p* < 0.05.

**TABLE 1:** Correlation between the plasma level of HIF3A and the clinical information of the LUAD patients

	LUAD (*n* = 103)		*p*‐Value
	Plasma HIF3A Low (*n* = 52)	Plasma HIF3A High (*n* = 51)	
Age (Years)			0.6058
≥60	29	31	
<60	23	20	
Gender			0.7391
Male	32	33	
Female	20	18	
Differentiation			0.6148
Well‐moderate	27	29	
Poor	25	22	
Tumor size			0.0005
≥5 cm	33	15	
<5 cm	19	36	
Lymph node metastasis			0.0384
Negative	18	28	
Positive	34	23	
Smoking history			0.7753
Yes	30	28	
No	22	23	

**TABLE 2:** Correlation between the plasma level of HIF3A and the clinical information of the LUSC patients

	LUSC (*n* = 96)		*p*‐Value
	Plasma HIF3A Low (*n* = 53)	Plasma HIF3A High (*n* = 43)	
Age (Years)			0.9723
≥60	31	25	
<60	22	18	
Gender			0.6334
Male	32	28	
Female	21	15	
Differentiation			0.9044
Well‐moderate	29	20	
Poor	24	23	
Tumor size			0.0011
≥5 cm	35	14	
<5 cm	18	29	
Lymph node metastasis			0.0012
Negative	22	32	
Positive	31	11	
Smoking history			0.5473
Yes	34	25	
No	19	18	

## 3 RESULTS

### 3.1 Characteristics of patients

A total of 199 NSCLC patients were enrolled to evaluate HIF3A expression in plasma samples, consisting of 103 LUAD and 96 LUSC patients with higher or lower expression of HIF3A. Furthermore, the patients were characterized according to clinical parameters such as age, sex, smoking history, differentiation, tumor size, and lymph node (LN) metastasis. Among the LUAD and LUSC groups, the tumor size and LN metastasis were statistically significant compared with the other parameters (Table 1 and Table 2).

### 3.2 HIF3A expression in the tumor tissues and plasma of NSCLC patients

**FIGURE 1:**
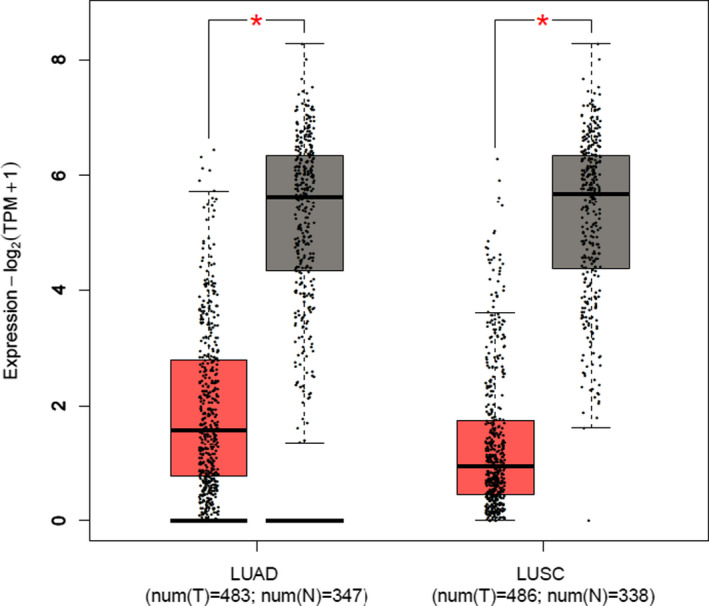
Bioinformatics‐based differential signature scores analysis in box plots by using GEPIA2 tool. The signature score is calculated by the mean value of log_2_ (TPM +1) of each gene in HIF3A signature gene set. The red box indicates the tumor samples, while the gray one represents the normal adjacent tissues. The HIF3A signature score level in NSCLC is significantly lower than normal tissues

**FIGURE 2:**
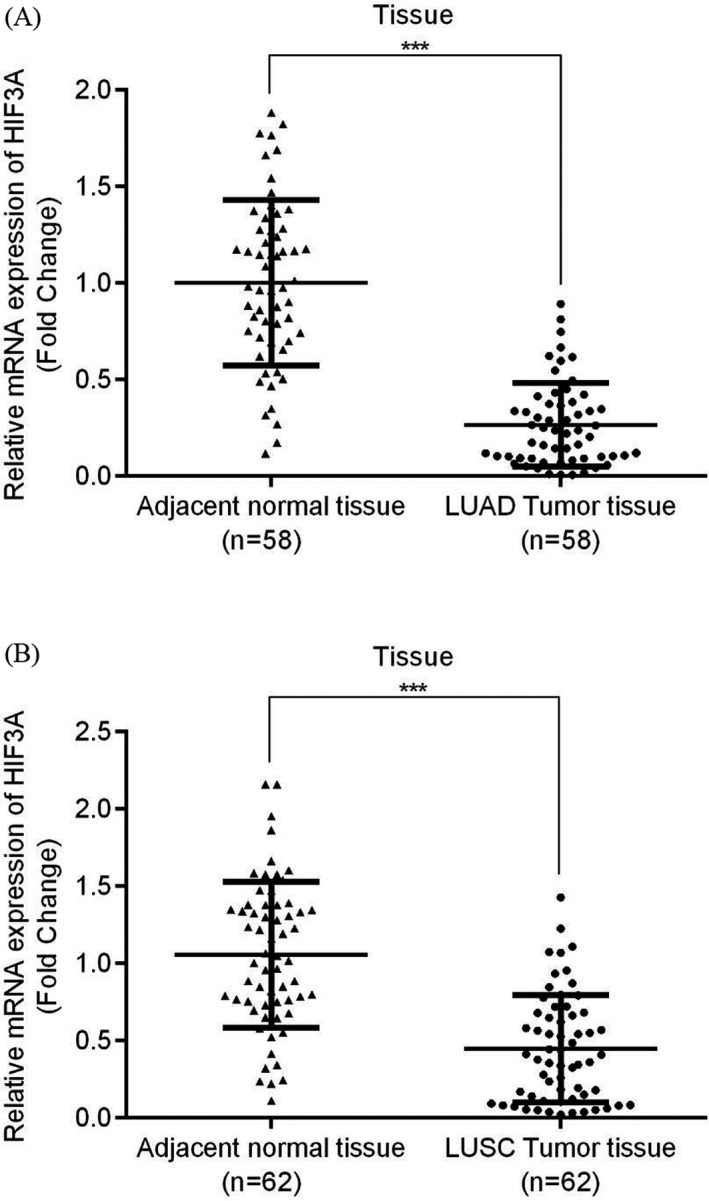
Expression of HIF3A in tumor and adjacent normal tissue of patients with NSCLC. In (A) LUAD and (B) LUSC groups, the relative HIF3A mRNA was significantly downgraded expression between tumor tissues compared with adjacent normal tissues; ***p < 0.001

**FIGURE 3:**
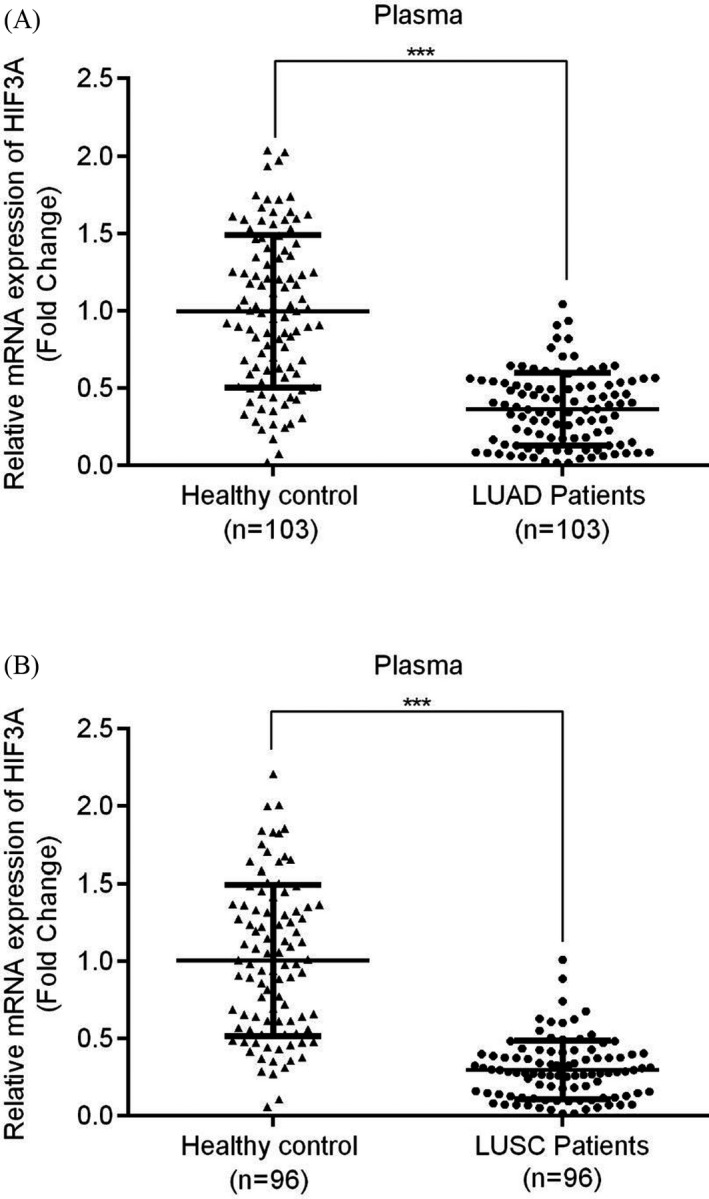
Expression of HIF3A in plasma samples of patients with NSCLC. (A) LUAD (B) LUSC both groups have significantly lower expression of HIF3A; ***p < 0.001

Bioinformatics tools were used to analyze HIF3A expression between tumor tissues and their adjacent normal tissues in LUAD and LUSC patients via signature score analysis (GEPIA 2). Overall, the results demonstrated that HIF3A expression levels in both groups were significantly lower in tumor tissues than in adjacent normal tissues (Figure 1, *p* < 0.05). Furthermore, in 58 patients with LUAD, the tumor tissues had significantly low expression of HIF3A mRNA (Figure 2A). Similarly, the expression of HIF3A mRNA was significantly diminished in the tumor tissues of 62 LUSC patients (Figure 2A), and HIF3A mRNA expression was significantly lower in plasma samples from 103 LUAD patients and 96 LUSC patients compared with healthy recruits (Figure 3A,B). Thus, HIF3A expression was significantly downregulated in the tumor tissues or plasma samples of NSCLC patients.

### 3.3 Correlation of HIF3A between plasma and tumor tissues

**FIGURE 4:**
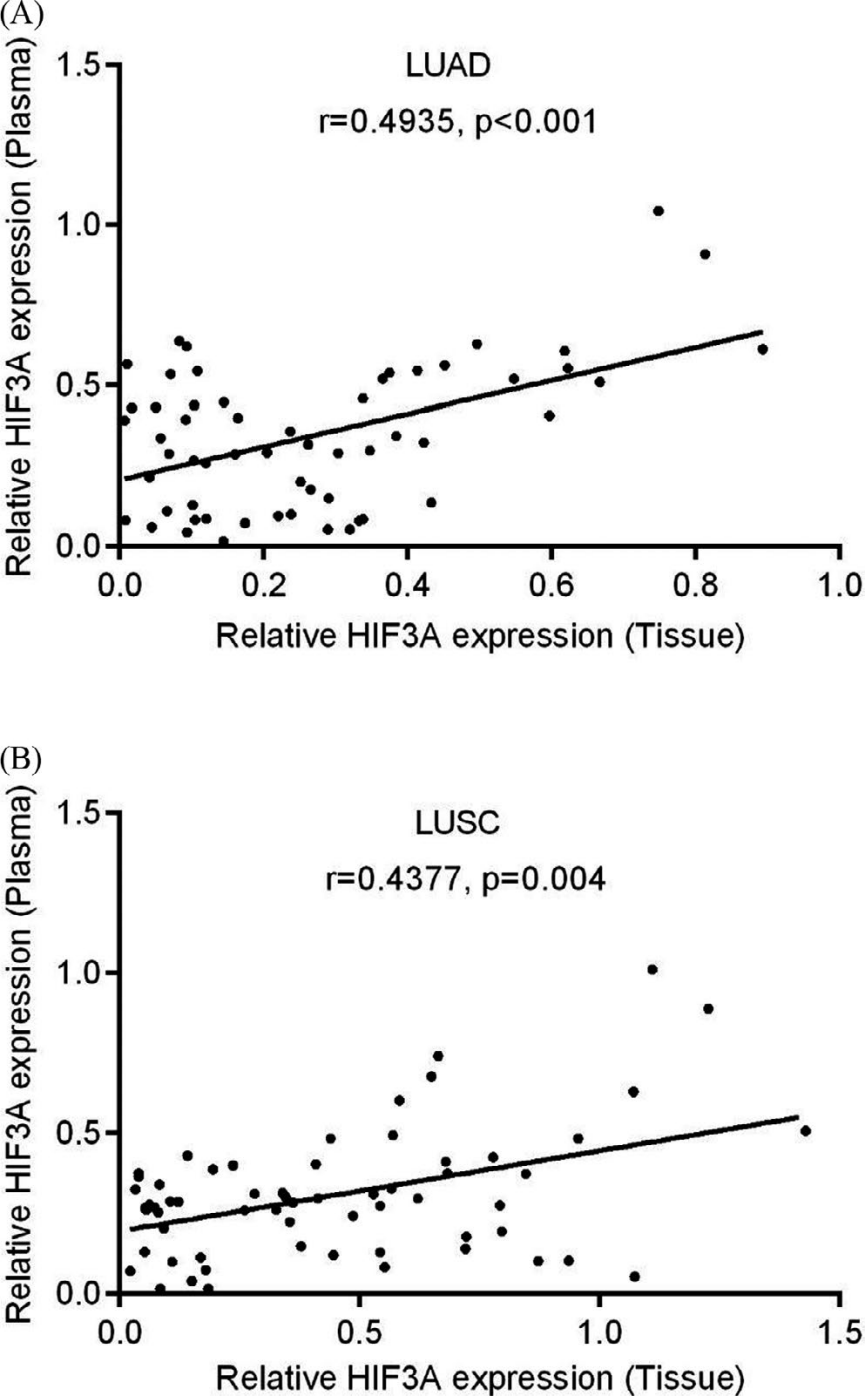
Correlation analysis between plasma and tissue expression level of HIF3A in NSCLC. (A) LUAD and (B) LUSC groups represent the positive and significant correlation between plasma and tumor tissue expression of HIF3A

The correlation between plasma and tumor tissues with respect to HIF3A expression in 58 LUAD and 62 LUSC patients is shown by scatter plots. Here, the LUAD patients showed a significantly moderate positive relationship between plasma and tumor tissues for HIF3A expression (Figure 4A; *r* = 0.493, *p* < 0.001). Subsequently, a significant positive correlation was observed between the plasma and tumor tissues in LUSC patients (Figure 4B; *r* = 0.437, *p* < 0.004). Therefore, the expression level of HIF3A was directly correlated between the plasma and tumor tissues of NSCLC patients.

### 3.4 Efficacy of HIF3A in NSCLC

**FIGURE 5:**
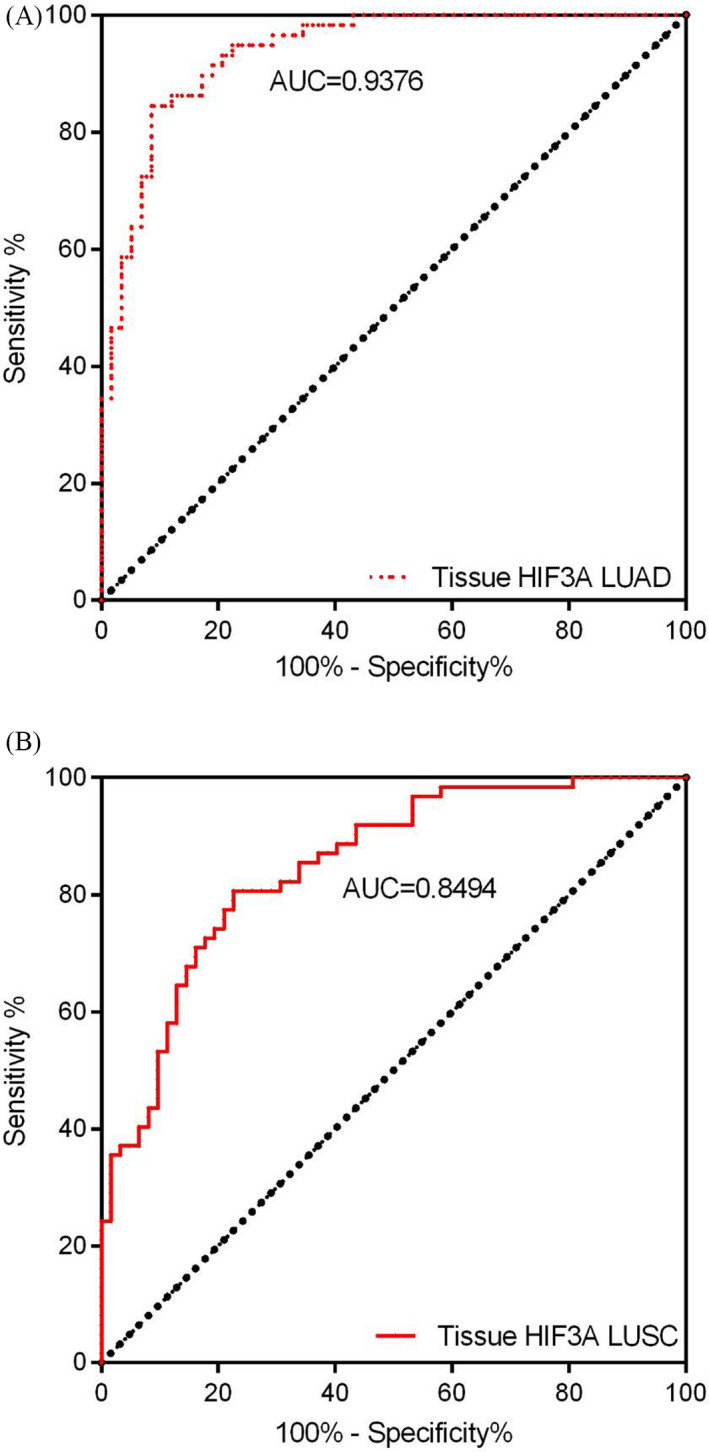
ROC curves of tumor tissue‐based HIF3A for patients with NSCLC. (A) LUAD (B) LUSC groups observed significant potential area under the curves (AUCs), for the tumor tissue‐based HIF3A

**FIGURE 6:**
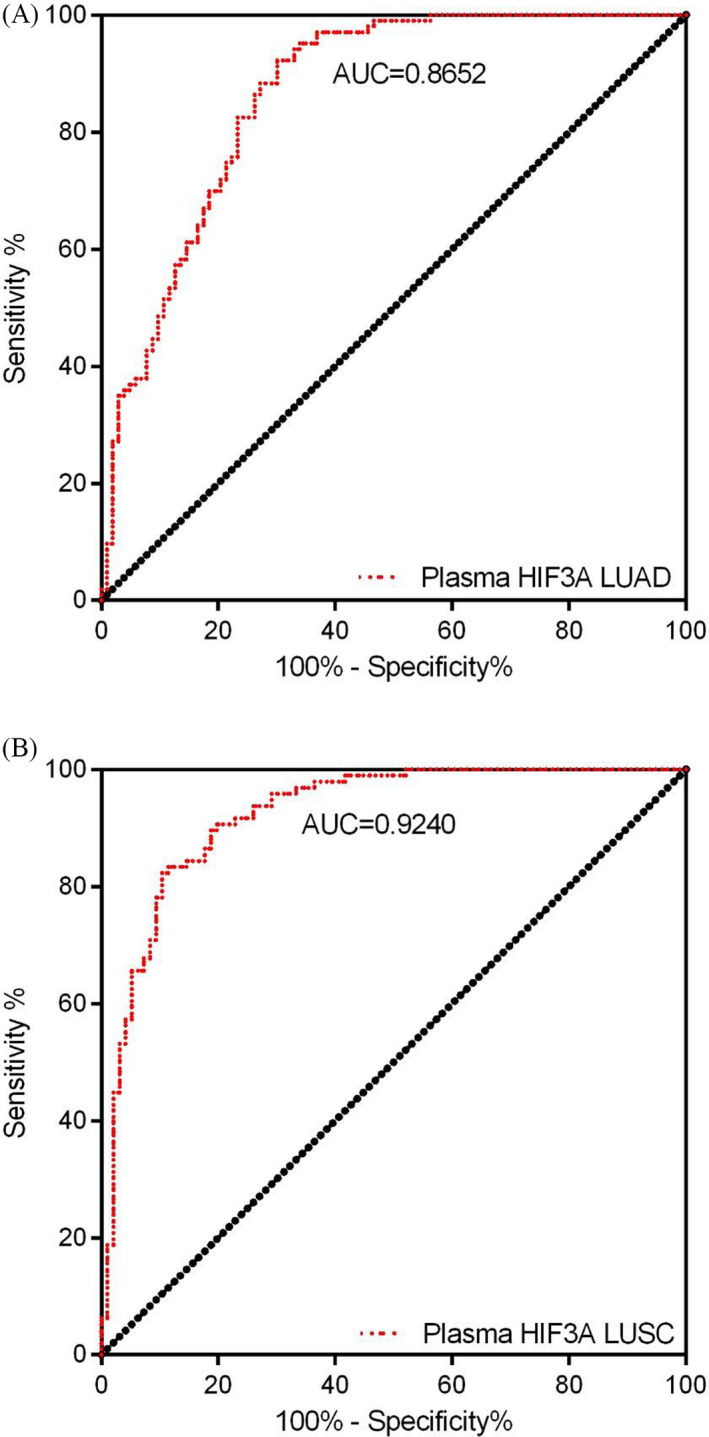
ROC of plasma‐based HIF3A for patients of NSCLC. (A) LUAD and (B) LUSC groups have achieved significant AUCs for plasma‐based HIF3A

The efficacy of detection of HIF3A was reported using the ROC‐AUC phenomenon in the tumor tissues and plasma samples of LUAD and LUCS patients. First, the tumor tissues with HIF3A expression in LUAD patients significantly achieved an AUC of 0.9376 (95% CI = 0.8961–0.9791, cutoff value 0.4594, specificity 91.38%, and sensitivity 84.48%) (Figure 5A), whereas in LUSC tumor tissues, AUC was 0.849 (95% CI = 0.7832–0.9155, cutoff value 0.7266, specificity 77.42%, and sensitivity 80.65%) (Figure 5B). In this context, HIF3A expression showed higher detection efficiency in LUAD tumor tissues than in LUSC patients. Second, the plasma expression of HIF3A was observed to be significant with AUCs of 0.865 (95% CI = 0.8155–0.9149, cutoff value 0.5694, specificity 82.52%, and sensitivity 76.70%) and 0.924 (95% CI = 0.8863–0.9618, cutoff value 0.4310, specificity 89.58%, and sensitivity 82.29%) in LUAD and LUSC patients, respectively (Figure 6A,B). Conversely, the plasma samples showed a slightly lower AUC in the LUAD group than in the LUSC group. Overall, the plasma and tumor tissues of NSCLC patients demonstrated notable efficacies for the diverse detection of HIF3A mRNA.

## 4 DISCUSSION

The HIF3A gene is located on chromosome 19q13.2, which is 43 kb long and comprises 19 exons.^14^, ^15^ To date, ten diverse HIF3A transcripts have been observed.^14^, ^15^, ^16^, ^17^, ^18^ It was previously discovered that human HIF‐3 α 1 expression was induced by hypoxia^19^ and that the HIF‐3 α 4 variant was downregulated.^15^, ^16^ Evaluation of HIF3A‐regulated genes has considerably progressed in recent years. One transcriptomic study reported HIF3A expression in the lung epithelium of a transgenic mouse line. The study observed many upregulated genes that were linked to the development of lung, while 25 genes were downregulated by DNA microarray analysis of lung RNA. The authors further provided evidence that in cultured A549 cells, SOX2 was a direct target gene of HIF3A.^20^ Thus, HIF3A may exist in the lung epithelium and play a role in the hypoxic environment of cancer cells or adjacent normal cells, in association with other targeted genes.

Human HIF3A may play an opposing role to HIF‐1 and HIF‐2, which have a significant role in the development and progression of cancers. Jang et al.^21^ reported that human HIF‐3 α 4 blocks vascular endothelial growth factor (VEGF) expression by inducing hypoxia, and hindering cell migration and tube formation in human umbilical vein endothelial cells, thereby repressing the process of new blood vessel growth (angiogenesis). Meanwhile, Maynard et al.^16^, ^17^ found that the HIF3A variant was downregulated in primary clear cell renal cell carcinomas and that von Hippel‐Lindau (VHL) gene mutations activated the hypoxia response pathway, and further exogenous expression of HIF‐3 α 4 variants suppressed the advancement of VHL‐null renal cell carcinoma. Similarly, a previous study reported that variants of HIF3A were detected at an identifiable level in the cDNA panels of cancer cells and subsequently showed that the HIF3A‐based three alternative first exons were discovered at a downgraded extent in multiple cancer cell lines.^18^ However, there have been no previous reports on HIF3A mRNA expression in NSCLC. In this study, we first investigated HIF3A mRNA gene expression in NSCLC‐based plasma and tissue samples using RT‐PCR assays. The results showed that the mRNA expression of the HIF3A gene was significantly downregulated in the NSCLC‐based plasma or tissue samples compared with that in healthy controls and adjacent noncancerous tissues. Our study findings are consistent with the above‐mentioned studies in that HIF3A expression was downregulated in plasma or tumor tissue samples.

The exact molecular mechanisms of HIF3A in human cancer, specifically in NSCLC, remain unknown. HIF1 α and HIF2 α affect tumor progression by directly regulating distinctive and allocated target genes such as SCGB3A1 in NSCLC,^22^ and ADRP, ADAM1, BNIP3, CCND1, GLUT1, IL‐6, TGF α, and VEGF in renal cell carcinoma.^4^, ^23^, ^24^, ^25^, ^26^ HIF α proteins also influence tumor progression by applying definite and often contrasting effects on critical oncoproteins and tumor suppressors, including p53, MYC, and mTOR.^4^ Thus, our study may indicate that HIF3A could be involved in tumor formation, occurrence, and progression of NSCLC, and subsequent studies are needed to fully understand and evaluate this hypothesis. Moreover, the present study explored the correlation between plasma and tissue samples for HIF3A expression, and they were positively and significantly correlated with each other (*p* < 0.05).

In recent years, the prognosis and survival rates of NSCLC patients are closely associated with clinical tumor stages, representing a keen drop from approximately 70%–90% in early‐stage (I/II) patients to 5%–10% in patients with advanced stages (III/IV).^27^, ^28^ The soaring mortality rate is chiefly due to the lack of influential steps for early detection and diagnosis, as only 15% of lung cancers are determined or diagnosed at early stages.^29^ Thus, wide‐ranging endeavors are vital for improving the efficacy of early detection and diagnosis to improve overall survival. Furthermore, this study evaluated the diagnostic value of HIF3A using the ROC curve, which achieved a significantly higher AUC of 0.84~0.93, in plasma and tissue samples of LUAD and LUSC. Taken together, our data suggest that HIF3A might be a promising therapeutic or diagnostic target gene for the treatment of NSCLC.

Currently, there are no related studies that can prove the diagnostic value of HIF3A in LUAD or LUSC, and as this study had a smaller sample size, to confirm our findings, a larger number of samples are required. The present study did not record whether patients received any chemotherapy or radiotherapy treatments; therefore, subsequent validation is essential. HIF3A could act as a novel target to be comprehensively studied in hypoxic cancer biology. Further studies are needed to increase our understanding of the role of HIF3A in the occurrence and development of NSCLC.

## 5 CONCLUSION

Hypoxia‐inducible factor 3 alpha is aberrantly detectable in plasma and tumor tissues of NSCLC patients and is a potentially discoverable target gene that might play an effective role in LUAD and LUSC. HIF3A expression was notably downregulated in the plasma or tumor tissues of LUAD and LUSC patients compared with the plasma of the healthy control group or adjacent tumor tissues. The present study validated the expression of HIF3A and its clinical significance in NSCLC. HIF3A may be involved in hypoxic responses during tumor occurrence and development.

## CONFLICT OF INTEREST

The authors declare that they have no potential conflicts of interest.

## Data Availability

The datasets used and analyzed during the current study are available from the corresponding author on reasonable request.
